# Developmental and molecular response of bovine embryos to reduced nutrients *in vitro*

**DOI:** 10.1530/RAF-20-0033

**Published:** 2020-12-23

**Authors:** Jason R Herrick, Sandeep Rajput, Rolando Pasquariello, Alison Ermisch, Nicolas Santiquet, William B Schoolcraft, Rebecca L Krisher

**Affiliations:** 1 Colorado Center for Reproductive Medicine, Lone Tree, Colorado, USA

**Keywords:** blastocyst, metabolism, fatty acid oxidation, AMPK, mTOR

## Abstract

Recent studies in our laboratory have indicated that bovine embryos only use a small amount of the nutrients available to them in culture. Our objective was to evaluate the developmental and molecular response of bovine embryos when nutrient concentrations in the culture medium were significantly reduced. Following IVM and IVF, embryos were cultured in media containing 75, 50, and 25% (experiment 1) or 25, 12.5, and 6.25% (experiment 2) of the concentrations of nutrients (carbohydrates, amino acids, and vitamins) present in our control medium (100%). Blastocyst formation, hatching, and allocation of cells to the inner cell mass (ICM) and trophectoderm (TE) were evaluated on day 7. Although the number of TE cells was decreased (*P* < 0.05) when nutrient concentrations were ≤25% (73.8–124.1 cells), it was not until nutrient concentrations were reduced to 6.25% that blastocyst formation (18.3 ± 3.0%) and hatching (3.0 ± 1.3%) were inhibited (*P* < 0.05) compared to embryos cultured in the control medium (156.1 ± 14.1 cells, 40.0 ± 3.8%, 20.0 ± 3.1%, respectively). Inhibition of fatty acid oxidation (etomoxir) reduced (*P* < 0.05) blastocyst development, with more pronounced effects at lower nutrient concentrations (≤12.5%). Reducing nutrient concentrations was associated with increased activity of AMPK, decreased activity of mTOR, and altered abundance of transcripts for hexokinase 1 (*HK1*), carnitine palmitoyl transferase 2 (*CPT2*), lactate dehydrogenase A (*LDHA*), and pyruvate dehydrogenase kinase 1 (*PDK1*), consistent with an increase in glucose and fatty acid metabolism. Reduced nutrient conditions provide a unique perspective on embryo metabolism that may facilitate the optimization of culture media.

**Lay summary:**

To support early embryo development in the first week after fertilisation, an appropriate mixture of nutrients (carbohydrates, amino acids, and vitamins) is needed in the culturing solution. However, refining these solutions to support optimal embryo health remains challenging. In this study, bovine (cow) embryos derived from abattoir material were used as a model for the development of other mammalian embryos, including humans. These embryos were cultured in the presence of 75, 50, 25, 12.5, or 6.25% of the nutrients present in control conditions (100%), which are similar to those reported for the fluids of the fallopian tubes and uterus. Embryo development was largely unaffected in the 75, 50, and 25% treatments, with some embryos developing in the presence of only 6.25% nutrients. Cow embryos are remarkably resilient to reduced concentrations of nutrients in their environment because they can utilize internal stores of fat as a source of energy.

## Introduction

Identification of an appropriate mixture of nutrients (what nutrients are present, what concentrations are used, and when those nutrients are provided) is a critical step in the process of formulating effective culture media for embryos. It is now well established that the concentration of carbohydrates, amino acids, and lipids present in the embryo’s environment not only affect the activity of metabolic pathways and ATP production, but also influence a wide variety of cellular functions that determine the viability of the resulting blastocysts and have implications for fetal development and offspring health (Fleming *et al.* 2004, Lane & Gardner 2005, Sinclair & Watkins 2013, Gardner & Harvey 2015). Recent studies have also shown that embryo metabolism plays a critical role in lineage specification, the maintenance of pluripotency, and epigenetic modifications (Harvey *et al.* 2016, Mathieu & Ruohola-Baker 2017, Chi *et al.* 2020, Hussein *et al.* 2020). Importantly, changes in maternal nutrition during gestation have been linked to the health of offspring, known as the Developmental Origins of Health and Disease, indicating the importance of embryo nutrition during both *in vitro* and *in vivo* development (Barker 2007, Mossa *et al.* 2013).

Given the large body of literature on the importance of nutrients and metabolic activity on embryo development, one might expect that embryos will only develop in media containing a specific set of nutrients present in a narrow range of concentrations. On the contrary, embryos will develop into blastocysts in a surprisingly large variety of media with dramatically different nutrient concentrations (Liu & Foote 1995, Holm *et al.* 1999, Gandhi *et al.* 2000, Lane *et al.* 2003). In some cases, empirical studies of medium composition directly contradict studies of the composition of the fluids of the maternal reproductive tract and the metabolic activity of embryos. For example, despite the presence of glucose in the bovine oviduct and uterus (Hugentobler *et al.* 2008) and studies showing significant consumption and metabolism of glucose (Rieger & Guay 1988, Thompson *et al.* 1996), some studies have shown that development of bovine embryos is actually improved when glucose is omitted from the culture medium (Takahashi & First 1992, Holm *et al.* 1999). This is not to say that embryo viability is equivalent in all culture conditions. There are developmental consequences of culturing embryos in sub-optimal culture conditions for even a short period (Lane & Gardner 1998). However, identifying optimal culture conditions for embryo development remains challenging.

Recent work in our laboratory has utilized a gas chromatography–mass spectrometry platform to analyze the nutrient composition of culture media following incubation of individual embryos to better characterize the metabolic profile of murine, bovine, feline, and human embryos (Krisher *et al.* 2015, Herrick *et al.* 2016*a*, 2018). Importantly, these analyses were performed with standard culture media and not media specifically designed for metabolic assessments. Therefore, our data are representative of metabolic activity during a normal culture period and not confounded by changes in medium formulation that are often made to simplify the analysis or interpretation of metabolic data. Results from these studies indicate that embryos consume only a small proportion of the nutrients available to them in culture. For bovine embryos, aspartate was the only nutrient of the 22 analyzed whose concentration decreased by >20% as a result of consumption by the embryos during culture (Krisher *et al.* 2015, Herrick *et al.* 2016*a*). For many of the other nutrients, including those known to be metabolized by bovine embryos (glucose, pyruvate, lactate), only a small (<5%) proportion of the nutrients present in the medium was actually consumed by the embryos. Although secretion and consumption of the same nutrients by the embryos could mask true rates of nutrient flux, our results are consistent with reports of carbohydrate and amino acid uptake and production in bovine embryos from other labs using alternative analytical techniques. For example, Thompson *et al.* (1996) reported that bovine morulae take up 11 pmol glucose/h and blastocysts take up 15 pmol glucose/h. Based on this rate, a single bovine embryo would be expected to consume 520–720 pmol of glucose during a 48 h incubation. If that embryo was cultured for 48 h in a 10 µL drop of medium containing 0.5–3 mM glucose (5000–30,000 pmol), similar to the conditions used in our previous studies (Krisher *et al.* 2015, Herrick *et al.* 2016*a*), consumption of 720 pmol of glucose would be 2.4–14.4% of the available glucose.

The small amount of nutrients being consumed relative to the amount of nutrients available in our bovine embryo culture system led us to hypothesize that the concentrations of nutrients present in the culture medium could be dramatically reduced and still support embryo development. A recent study by Ermisch *et al.* (2020) indicated that the concentrations of nutrients present during culture of murine embryos could be reduced by 50% with minimal effects on embryo development. However, reduction of nutrients, particularly pyruvate and lactate, by more than 50% severely compromised embryo development and viability. The current study was initiated to determine if bovine embryos could also successfully develop *in vitro* with significantly reduced nutrient concentrations and if the response of the bovine embryo to reduced nutrient availability was different than that observed in the mouse given the increased size, lipid content, and metabolic activity of the bovine embryo relative to that of the mouse (Gardner & Leese 1986, Houghton *et al.* 1996, Thompson *et al.* 1996, Genicot *et al.* 2005, Wildt *et al.* 2010). Specifically, the concentrations of all nutrients present in the medium were serially diluted from 100 to 6.25% to determine effects on blastocyst formation, hatching, and allocation of cells to the inner cell mass and trophectoderm. In the presence of severely reduced (<50%) nutrient concentrations, the relative role of endogenous lipid metabolism for embryo development was evaluated, as were the molecular responses of the embryo. Our results indicate that the remarkable resilience of the bovine embryo, relative to the murine embryo, to dramatic reductions in nutrient availability is associated with its ability to utilize endogenous lipids.

## Materials and methods

### Chemicals and laboratory supplies

Unless specified otherwise, all reagents were purchased from Sigma-Aldrich. All consumables (pipette tips, petri dishes, etc.) were screened for embryotoxicity using a mouse embryo assay prior to use (Herrick *et al.* 2016*b*). All media were prepared in our laboratory, filtered (0.22 μm; MillexGV, EMD Millipore), and equilibrated for at least 4 h before use to achieve a final, equilibrated pH of 7.25 ± 0.05. Unless stated otherwise, all cultures were performed in media covered with oil (OvOil, Vitrolife, Englewood, CO, USA) in tissue-culture treated Petri dishes (Primaria™, Corning) in incubators maintained at 38.7°C with maximal humidity.

### In vitro maturation

Bovine cumulus-oocyte complexes (COCs) were recovered from abattoir-derived ovaries by a commercial company (DeSoto Biosciences) and matured in a defined medium (Herrick *et al.* 2016*a*) containing recombinant human EGF (50 ng/mL), recombinant human follicle-stimulating hormone (Follistim, Merck & Co., Inc; 0.1 IU/mL), recombinant human hyaluronan (0.125 mg/mL, Novozymes, Bagsvaerd, Denmark), and recombinant human albumin (2.5 mg/mL, AlbIX, Novozymes). Groups of 50 COCs were matured in 2 mL of medium in sealed tubes gassed with 5% CO_2_ in air and maintained at ~38.5°C in a portable incubator during overnight shipment to our laboratory.

### In vitro fertilization

Following 22–23 h of maturation, COCs were recovered from the shipping tubes, washed, and transferred in groups of 10 into 45 µL drops of fertilization medium containing 2.0 mM caffeine, 7.5 µg/mL heparin, and 8.0 mg/mL fatty acid free (FAF) BSA (MP Biomedicals, Solon, OH, USA) (Herrick *et al.* 2016*a*). Cryopreserved spermatozoa from a single bull were thawed and centrifuged (20 min, 1200 ***g***, 22°C) through a discontinuous (45%:90%) gradient of PureSperm (Nidacon, Mölndal, Sweden). The resulting sperm pellet was washed twice (5 min, 600 ***g***, 22°C) in a MOPS-buffered medium and then diluted with fertilization medium. Spermatozoa were added to drops containing COCs for a final concentration of 2 × 10^6^ spermatozoa/mL and gametes were co-incubated in 7.5% CO_2_ in air at 38.7°C for 20–22 h. The concentration of CO_2_ is increased to compensate for the elevation of our laboratory (~1830 m above sea level) and is approximately equal to 6.0% CO_2_ at sea level (media pH 7.25 ± 0.05).

### *In vitro* embryo culture

Remaining cumulus cells and loosely bound spermatozoa were removed from presumptive zygotes by shaking on a vortex mixer for 2.5 min. Presumptive zygotes were washed once in our standard IVC1 medium (100% bovine Optimized Embryo Culture 1 (bOEC1), Table 1 (Herrick *et al.* 2016*a*)), randomly allocated to treatments, and then washed twice in the treatment medium before placing in culture. During the first 72 h of culture, embryos were cultured in groups of 10 in 20 µL drops of medium. On day 3 (72 h in bOEC1, 96 h post-insemination), cleavage to at least the two-cell stage was evaluated and embryos with more than four cells were washed, transferred to fresh medium (bOEC2, Table 2 (Herrick *et al.* 2016*a*)), and cultured in groups of 5 in 20 µL drops. In all experiments, embryos were treated (e.g. dilution of nutrients or the presence of etomoxir) the same in both steps of culture. On day 7 of culture (96 h in bOEC2, 192 h post-insemination), blastocyst formation and hatching were evaluated.

**Table 1:** Nutrient concentrations (mM) in various dilutions (100–6.25%) of bovine optimized embryo culture medium 1 (bOEC1, 0–72 h of culture), as well as those reported for bovine oviductal fluid and culture media used by other laboratories for bovine embryo culture.

	bOEC1						*In vivo* oviduct^1^	Synthetic oviduct fluid				CDM1^6^	KSOM^7^	G1.2^8^
	100%	75%	50%	25%	12.5%	6.25%		Original^2^	Modified^3^	Modified^4^	Sequential^5^			
Glucose	0.500	0.375	0.250	0.125	0.063	0.031	2.59	1.5	0	0	1.5	0.5	0.2	0.5
Pyruvate	0.300	0.225	0.150	0.075	0.038	0.019	0.11	0.33	0.3	7.27	0.33	0.5	0.2	0.32
l-lactate	10.000	7.500	5.000	2.500	1.250	0.625	5.93	3.3	3.3	5.35	3.3	10	10	10.5
Citrate	0.500	0.375	0.250	0.125	0.063	0.031		0	0	0.34	0	0.5	0	0.5
Ala-Gln	1.000	0.750	0.500	0.250	0.125	0.063	0.19	0	1	0.2	1	1	1	0.5
Taurine	0.100	0.075	0.050	0.025	0.013	0.006	0.05	0	0	0	0.1	0.1	0	0.1
NEAA														
Ala	0.100	0.075	0.050	0.025	0.013	0.006	0.60	0	0.1	0.05	0.1	0.1	0.1	0.1
Asn	0.100	0.075	0.050	0.025	0.013	0.006	0.04	0	0.1	0.05	0.1	0.1	0.1	0.1
Asp	0.100	0.075	0.050	0.025	0.013	0.006	0.14	0	0.1	0.05	0.1	0.1	0.1	0.1
Glu	0.100	0.075	0.050	0.025	0.013	0.006	0.35	0	0.1	0.05	0.1	0.1	0.1	0.1
Gly	1.100	0.825	0.550	0.275	0.138	0.069	1.48	0	0.1	0.05	0.1	5	0.1	0.1
Pro	0.100	0.075	0.050	0.025	0.013	0.006		0	0.1	0.05	0.1	0.1	0.1	0.1
Ser	0.100	0.075	0.050	0.025	0.013	0.006	0.17	0	0.1	0.05	0.1	0.1	0.1	0.1
EAA														
Arg	0.150	0.113	0.075	0.038	0.019	0.009	0.13	0	0.1	0.1	0.6	0	0.3	0
Cys	0.025	0.019	0.013	0.006	0.003	0.002		0	0.05	0.05	0.1	0	0.05	0
His	0.050	0.038	0.025	0.013	0.006	0.003	0.07	0	0.05	0.05	0.2	0	0.1	0
Iso	0.100	0.075	0.050	0.025	0.013	0.006	0.08	0	0.2	0.2	0.4	0	0.2	0
Leu	0.100	0.075	0.050	0.025	0.013	0.006	0.19	0	0.2	0.2	0.4	0	0.2	0
Lys	0.100	0.075	0.050	0.025	0.013	0.006	0.23	0	0.2	0.2	0.4	0	0.2	0
Met	0.025	0.019	0.013	0.006	0.003	0.002	0.04	0	0.05	0.05	0.1	0	0.05	0
Phe	0.050	0.038	0.025	0.013	0.006	0.003	0.07	0	0.1	0.1	0.2	0	0.1	0
Thr	0.100	0.075	0.050	0.025	0.013	0.006	0.15	0	0.2	0.2	0.4	0	0.2	0
Trp	0.013	0.009	0.006	0.003	0.002	0.001	0.04	0	0.02	0.02	0.05	0	0.025	0
Tyr	0.050	0.038	0.025	0.013	0.006	0.003	0.05	0	0.1	0.1	0.2	0	0.1	0
Val	0.100	0.075	0.050	0.025	0.013	0.006	0.17	0	0.2	0.2	0.4	0	0.2	0
Total nutrients (mmol/L)	14.96	11.22	7.48	3.74	1.87	0.94	12.86	5.13	6.77	14.98	10.38	18.20	13.83	13.12

^1^Hugentobler *et al.* (2007, 2008); ^2^Tervit *et al.* (1972); ^3^Takahashi and First (1992); ^4^Holm *et al.* (1999); ^5^Gandhi *et al.* (2000); ^6^Barcelo-Fimbres and Seidel (2007) and De La Torre-Sanchez *et al.* (2006); ^7^Liu and Foote (1995); ^8^Lane *et al.* (2003).

**Table 2:** Nutrient concentrations (mM) in various dilutions (100–6.25%) of bovine optimized embryo culture medium 2 (bOEC2, 72–168 h of culture), as well as those reported for bovine uterine fluid and culture media used by other laboratories for bovine embryo culture.

	bOEC2						*in vivo* uterus^1^	Synthetic oviduct fluid				CDM2^6^	KSOM^7^	G2.2^8^
	100%	75%	50%	25%	12.5%	6.25%		Original^2^	Modified^3^	Modified^4^	Sequential^5^			
Glucose	0.000	0.000	0.000	0.000	0.000	0.000	3.90	1.5	0	0	3	0	0.2	3.15
Fructose	3.000	2.250	1.500	0.750	0.375	0.188		0	0	0	0	2	0	0
Pyruvate	0.100	0.075	0.050	0.025	0.013	0.006	0.11	0.33	0.3	7.27	0.33	0.2	0.2	0.1
l-lactate	6.000	4.500	3.000	1.500	0.750	0.375	1.06	3.3	3.3	5.35	3.3	5	10	5.87
Citrate	0.500	0.375	0.250	0.125	0.063	0.031		0	0	0.34	0	0.5	0	0.5
Ala-Gln	1.000	0.750	0.500	0.250	0.125	0.063	0.19	0	1	0.2	1	1	1	1
Taurine	0.100	0.075	0.050	0.025	0.013	0.006	0.52	0	0	0	0	0	0	0
NEAA														
Ala	0.100	0.075	0.050	0.025	0.013	0.006	0.34	0	0.1	0.05	0.1	0.1	0.1	0.1
Asn	0.100	0.075	0.050	0.025	0.013	0.006	0.14	0	0.1	0.05	0.1	0.1	0.1	0.1
Asp	0.100	0.075	0.050	0.025	0.013	0.006	0.12	0	0.1	0.05	0.1	0.1	0.1	0.1
Glu	0.100	0.075	0.050	0.025	0.013	0.006	0.22	0	0.1	0.05	0.1	0.1	0.1	0.1
Gly	1.100	0.825	0.550	0.275	0.138	0.069	1.39	0	0.1	0.05	0.1	5	0.1	0.1
Pro	0.100	0.075	0.050	0.025	0.013	0.006		0	0.1	0.05	0.1	0.1	0.1	0.1
Ser	0.100	0.075	0.050	0.025	0.013	0.006	0.22	0	0.1	0.05	0.1	0.1	0.1	0.1
EAA														
Arg	0.300	0.225	0.150	0.075	0.038	0.019	0.18	0	0.1	0.1	0.6	0.1	0.3	0.6
Cys	0.050	0.038	0.025	0.013	0.006	0.003		0	0.05	0.05	0.1	0.05	0.05	0.1
His	0.100	0.075	0.050	0.025	0.013	0.006	0.12	0	0.05	0.05	0.2	0.05	0.1	0.2
Iso	0.200	0.150	0.100	0.050	0.025	0.013	0.09	0	0.2	0.2	0.4	0.2	0.2	0.4
Leu	0.200	0.150	0.100	0.050	0.025	0.013	0.19	0	0.2	0.2	0.4	0.2	0.2	0.4
Lys	0.200	0.150	0.100	0.050	0.025	0.013	0.20	0	0.2	0.2	0.4	0.2	0.2	0.4
Met	0.050	0.038	0.025	0.013	0.006	0.003	0.04	0	0.05	0.05	0.1	0.05	0.05	0.1
Phe	0.100	0.075	0.050	0.025	0.013	0.006	0.08	0	0.1	0.1	0.2	0.1	0.1	0.2
Thr	0.200	0.150	0.100	0.050	0.025	0.013	0.14	0	0.2	0.2	0.4	0.2	0.2	0.4
Trp	0.025	0.019	0.013	0.006	0.003	0.002	0.04	0	0.02	0.02	0.05	0.02	0.025	0.05
Tyr	0.100	0.075	0.050	0.025	0.013	0.006	0.06	0	0.1	0.1	0.2	0.1	0.1	0.2
Val	0.200	0.150	0.100	0.050	0.025	0.013	0.19	0	0.2	0.2	0.4	0.2	0.2	0.4
Total nutrients (mmol/L)	14.13	10.59	7.06	3.53	1.77	0.88	9.49	5.13	6.77	14.98	11.78	15.77	13.83	14.77

^1^Hugentobler *et al.* (2007, 2008); ^2^Tervit *et al.* (1972); ^3^Takahashi and First (1992); ^4^Holm *et al.* (1999); ^5^Gandhi *et al.* (2000); ^6^Barcelo-Fimbres and Seidel (2007) and De La Torre-Sanchez *et al.* (2006); ^7^Liu and Foote (1995); ^8^Lane *et al.* (2003).

For each replicate (day of ovary collection) a concentrated stock solution of salts (NaCl, KCl, KH_2_PO_4_, CaCl_2_-2H_2_O, MgSO_4_-7H_2_O, and NaHCO_3_), antibiotics (gentamicin, 25 µg/mL), macromolecules (hyaluronan, 0.125 mg/mL and FAF BSA, 2.5 mg/mL), and growth factors (insulin, transferrin, and selenium, ITS) was prepared (Herrick *et al.* 2016*a*). A separate concentrated stock solution of nutrients, defined as carbohydrates (glucose, fructose, pyruvate, l-lactate, and citrate), amino acids (glycine, alanyl-glutamine, taurine, NEAA, and EAA), vitamins, and EDTA, was also prepared. Although not a metabolite for ATP production, EDTA was included due to its reported effects on the rate of glycolysis (Lane & Gardner 2001). All working culture media contained the same amount of the salt solution and varying amounts of the nutrient solution. The difference in volumes was corrected by adding culture grade water so that final nutrient concentrations were 75, 50, and 25% (Experiment 1) or 25, 12.5, and 6.25% (Experiment 2; Tables 1 and 2) of the concentrations present in our laboratory’s standard medium (100%). The concentrations of NaCl (100 mM) and NaHCO_3_ (25 mM), which account for 250 mOsm of the total osmolarity, were the same in all media. The nutrients in our 100% (control) medium account for <15 mOsm of the total osmolarity of the medium. Differences in osmolarity between 250 and 265 mOsm do not affect the development of bovine embryos (Liu & Foote 1996). By simultaneously diluting all of the nutrients, the ratios between concentrations of different nutrients remained consistent to minimize effects on substrate transport and/or enzyme kinetics that may occur if these ratios were altered by diluting some nutrients and not others (Thompson *et al.* 1993, Gardner *et al.* 1994, Van Winkle & Dickinson 1995). The same dilution was used for both steps of culture, such that even if the specific concentrations of nutrients were different between steps 1 and 2 of culture, the dilution of those nutrients relative to the control (100%) was the same. Etomoxir (50 µM), an inhibitor of carnitine palmitoyltransferase 1 (Dunning *et al.* 2010, Paczkowski *et al.* 2013), was used to evaluate the importance of fatty acid oxidation (FAO) when nutrient concentrations were reduced. Stock solutions of etomoxir (10 mM) were prepared in culture grade water and stored at −80°C until used for medium preparation for each replicate.

### Allocation of cells to the trophectoderm and inner cell mass

Hatching and fully-hatched blastocysts were fixed for 20 min in 4% paraformaldehyde (Electron Microscopy Sciences, Hatfield, PA, USA) and then stored in PBS with 0.5% BSA (MP Biomedicals) until staining. After washing three times in PBS with 0.1% PVP and 0.1% Triton X-100 (TX100), blastocysts were permeabilized in PBS with 1.0% TX100 (30 min). Embryos were incubated in a blocking medium (PBS with 0.1% TX100, 0.1 M glycine, 0.5% BSA, and 10% (v/v) horse serum) for 2 h and then incubated with primary antibodies (18–24 h, 4°C) for sex-determining region Y-box 2 (SOX2; rabbit monoclonal, anti-human, AN579; Biogenex, Fremont, CA, USA) and caudal type homeobox 2 (CDX2; mouse monoclonal, anti-human, MU392A; Biogenex) (Bakhtari & Ross 2014, Herrick *et al.* 2016*a*). Following three washes in PBS with 0.1% PVP and 0.1% TX100, blastocysts were incubated (1 h) with secondary antibodies (Alexa Fluor 488 donkey anti-rabbit IgG (A-2126, SOX2; Thermo Fisher Scientific) and Alexa Fluor 555 goat anti-mouse IgG (A-21424, CDX2; Thermo Fisher Scientific)), washed three more times, and mounted on a glass slide in ProLong Diamond Antifade reagent containing DAPI (Life Technologies, Thermo Fisher Scientific). Cells were visualized using a fluorescent microscopy (Olympus BX52) and counted using the manual count function of MetaMorph software. Cells positive for SOX2 were considered ICM cells, cells positive for CDX2 were considered TE cells, and the total number of cells in the blastocyst was calculated as the sum of SOX2- and CDX2-positive cells.

### Embryo vitrification and warming

Based on the results of experiments 1 and 2, embryonic development began to be negatively affected (reduced trophectoderm cell numbers) when nutrient concentrations were reduced to 25% of our control medium. To further evaluate the viability of embryos produced in this medium, day 6 (72 h in IVC2, 168 h post-insemination) expanded and early hatching (<1/2 of the volume of the embryo exterior to the zona pellucida) blastocysts cultured with 100% or 25% nutrients were vitrified following the protocol for CryoTops (https://www.kitazato-dibimed.com/wp-content/uploads/VITRIFICATION-PROTOCOL.pdf) provided online by the Kitazato Corporation (Tokyo Japan). Briefly, blastocysts were equilibrated for 15 min at 22°C in a HEPES-buffered, Ca^+2^-free medium containing 1.0 mg/mL hydroxypropyl cellulose (HPC), 7.5% (v/v) ethylene glycol (EG), and 7.5% (v/v) dimethyl sulfoxide (DMSO) (Coello *et al.* 2016). Embryos were then incubated for 1 min in a vitrification solution containing 1.5 mg/mL HPC, 15% (v/v) EG, 15% (v/v) DMSO, 40.0 mg/mL Ficoll, and 0.6 M trehalose, loaded onto the tip of a CryoTop device in a minimal volume, and quickly submerged into liquid nitrogen (LN_2_). Cryotops were placed inside a protective sleeve and stored in LN_2_ for ~4 months. For warming, cryotops were removed from the protective sleeves while still submerged in LN_2_, quickly removed from the LN_2_, and the tip of the CryoTop submerged in 3 ml of warm (37°C) thawing solution (1.0 M trehalose and 20% (v/v) fetal calf serum, FCS). Embryos were then transferred to a dilution solution (0.5 M trehalose, 20% FCS) for 3 min, before washing (0.0 M trehalose, 20% FCS), and returning to culture. All embryos were cultured in the control medium (100%) in groups of 4–5 in 20 µL drops (38.7°C, 7.5% CO_2_, 5% O_2_) and evaluated for survival, re-expansion, and the completion of hatching (embryo completely outside of the zona pellucida) after 48 h of culture.

### Blastocyst gene expression

Expression of the following genes (Table 3) were analyzed in expanded and early hatching blastocysts that had been cultured in media with 50, 25, 12.5, or 6.25% nutrients: carnitine palmitoyltransferase 2 (*CPT2*), hexokinase 1 (*HK1*), lactate dehydrogenase A (*LDHA*), pyruvate dehydrogenase kinase 1 (*PDK1*), and prostaglandin-endoperoxide synthase 2/cyclooxygenase 2 (*PTGS2*). Data were normalized using expression of the housekeeping gene 18S rRNA (*RN18S1*). A total of three to four biological replicates (pools of 8 blastocysts) were used for quantitative real-time PCR (RT-qPCR) after snap freezing in liquid nitrogen in 10 µL PBS + 0.01 % PVP. RNA extraction was performed using the PicoPure RNA Isolation Kit (Thermofisher Scientific) with on-column DNase treatment (Qiagen). cDNA was synthesized using qScript™ cDNA Supermix (Quanta Biosciences, Gaithersburg, MD) following the manufacture’s protocol. The cDNA samples were diluted 1:5 using RNase free water and stored at −20°C until RT-qPCR was run. Each RT-qPCR reaction was performed in duplicate using 12.5 µL Power SYBR™ Green PCR Master Mix (Applied Biosystems), 2.5 µL 10 µM primer mix and 5 µL 1:5 diluted cDNA sample. The RT-qPCR program was: 50°C for 2 min for the first cycle and 95°C for 2 min for the second cycle followed by 40 cycles of amplification at 95°C for 10 s and 59°C for 1 min. A melting curve was analyzed for each experiment to assess the specificity of primer amplification. Relative gene expression was calculated using the 2^−ΔΔCt^ method (Pfaffl 2001). Normalization of Ct values was obtained using expression of *RN18S*.

**Table 3:** Primer sequences used for analysis of gene expression.

Gene ID	Accession number ID	Forward primer (5’ → 3’)	Reverse primer (5’ → 3’)
*CPT2*	NM_001045889	AGCAGATGATGGCCAAGTG	CAGGTACCGCAGAGCAAAC
*HK1*	NM_001012668	CTGCTTGACAAAGCCATCAA	CGTCGTAGCCACAGGTCAT
*LDHA*	NM_174099	GCAAGTTGCTTGTTGTTTCC	CAGATTGCAACCACTTCCA
*PDK1*	NM_001205957	GTCACCAGCCAGAATGTTCA	TCCGATGAGATAGGCTTCCT
*PTGS2*	NM_174445	AAGATCTCCTTCCTGCGAAA	ATCAGGCACAGGAGGAAGAG
*RN18S1*	AC_000182.1	CGGCGACGACCCATTCGAAC	GAATCGAACCCTGATTCCCCGTC

### Western blot analysis

Western blot analysis was used to determine the expression and phosphorylation level of target proteins. Groups of 20 expanded or early hatching blastocysts produced in media with 50% (the lowest concentration of nutrients with no significant effect on embryo development) or 6.25% nutrients were placed in 20 μL of radioimmunoprecipitation assay (RIPA) buffer (Millipore Sigma, R0278) containing cocktails of protease (Millipore Sigma, 11836170001) and phosphatase (78420; Thermo Fisher Scientific) inhibitors. Samples were then mixed with 6 μL of 4× Laemmli buffer and boiled at 100°C for 10 min to ensure complete embryo lysis and protein denaturation under reduced conditions. After a 5 min incubation at room temperature, protein samples were resolved on 4–20% Mini-Protean TGX Precast gels (Bio-Rad) and blotted onto a polyvinylidene fluoride (PVDF) membranes (Millipore; #IPVH00010). Blotted membranes were then incubated in blocking buffer (3% BSA in 1× TBST) at room temperature (RT) for 1 h and probed with primary antibodies against total mammalian target of rapamycin (t-mTOR), phosphorylated mTOR (phospho mTOR^(Ser2448)^), total AMP-activated protein kinase (t-AMPK), phosphorylated AMPK (phospho AMPK^(T172)^), total protein kinase B (t-AKT), and phosphorylated AKT (phospho AKT^(S473)^) (Cell Signaling Technology) at 1:2000 dilution and actin (Millipore Sigma, MAB1501) at 1:5000 in blocking buffer at 4°C overnight. Unbound primary antibodies were removed by washing three times for 3 min in 1× TBST before incubation with the appropriate secondary antibody, HRP anti-rabbit-IgG (Cell Signaling Technology, 7074S) or anti-mouse-IgG (Thermo Fisher Scientific, PA1-74421) at 1:5000 dilutions in blocking buffer at RT for 1 h. The membrane was then washed four times for 3 min in 1× TBST and immunoreactive protein bands were detected using Super Signal West Dura Chemiluminescent substrate (Thermo Fisher Scientific, 34076). Images were captured using ChemiDoc XRS (Bio-Rad) and subjected to quantitative densitometry analysis using ImageJ software (http://imagej.nih.gov/ij/). Total protein abundance was expressed relative to actin, and phosphorylation level of target proteins was determined based on the ratio of phosphorylated and total protein abundance in each sample.

### Statistical analysis

In each replicate (day of ovary collection), presumptive zygotes were randomly allocated to treatments in a complete block design. Embryonic development was analyzed using the generalized linear mixed model (GLIMMIX) procedure in SAS. The proportions of embryos developing to the blastocyst or hatching blastocyst stage was based on the number of cleaved embryos (≥2 cells at 96 h postinsemination). Each embryo was scored as a 1 or 0 depending on whether or not it achieved the desired stage of development (e.g. cleaved, blastocyst, or hatching blastocyst) and analyzed using an ANOVA model with a binomial error distribution and a probit link function. Treatment was included as a fixed factor and replicate was included as a random factor. Blastocyst cell numbers were analyzed using the mixed model procedures in SAS with treatment as the only fixed factor. Statistical analyses of RT-qPCR were completed using IBM SPSS (IBM) using one-way ANOVA (multiple comparisons). Western blot data were analyzed using Student’s t-test using GraphPad. Prism version 8.20. In all cases, results were considered statistically significant when P< 0.05. Unless otherwise stated, results are presented as mean ± s.e.m.

## Results

### Effectiveness and efficiency of the culture system

In experiments 1 and 2, 529 presumptive zygotes cultured in control (100% nutrients) conditions produced 342 cleaved (≥2 cells) embryos (64.7%) and the majority of these (*n* = 261, 76.3%) contained ≥4 cells on day 3 and were transferred into the second step of culture. Blastocyst formation occurred in 27.6% of presumptive zygotes or 42.7% of cleaved (≥2 cells) embryos. Hatching was observed in 14.6% of presumptive zygotes and 22.5% of cleaved (≥2 cells) embryos. Hatching or fully hatched (*n* = 70) blastocysts contained 191.0 ± 9.7 cells, with 155.4 ± 8.7 cells in the TE and 35.6 ± 2.4 cells in the ICM (19.2% of total cells).

### Experiment 1: 100, 75, 50, and 25% nutrients

**Figure 1:**
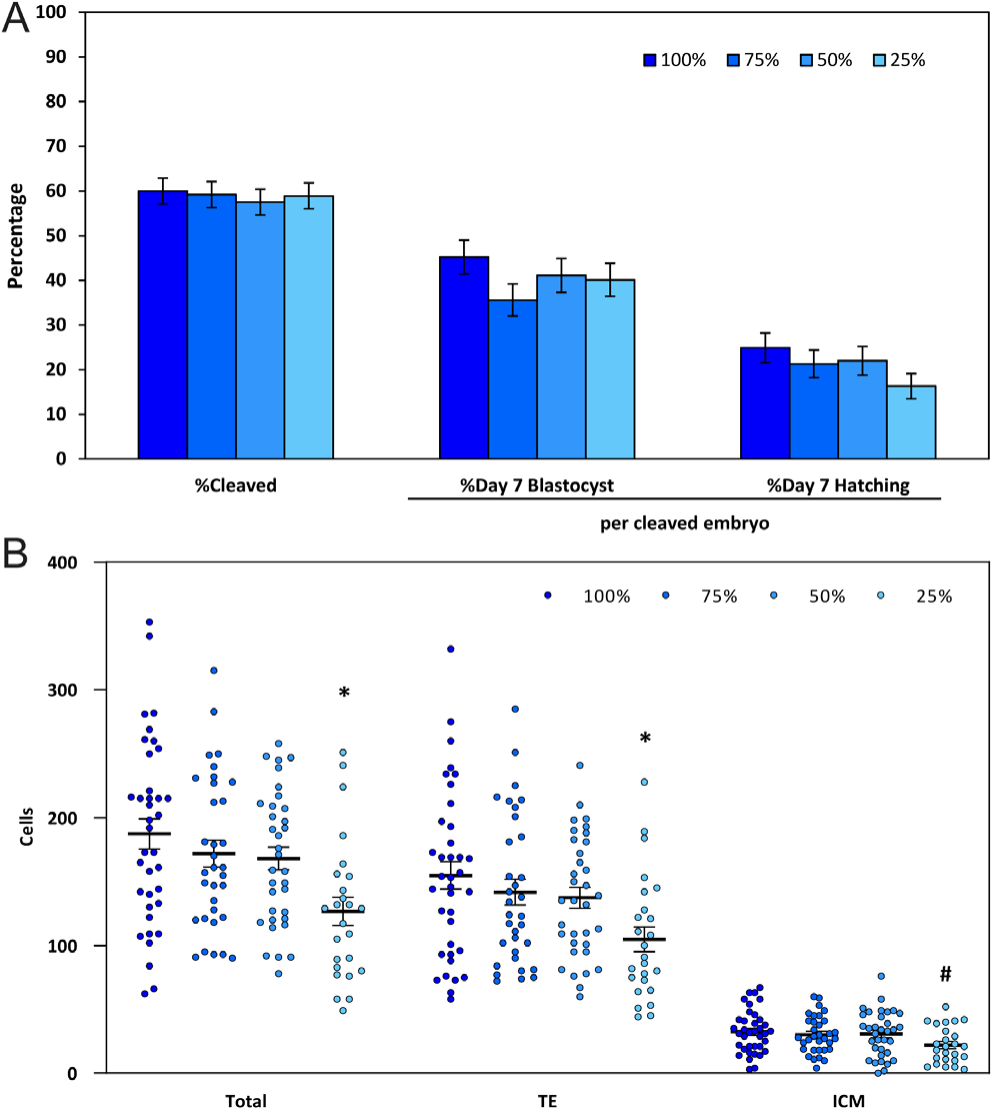
Development of embryos following culture in media containing 100, 75, 50, or 25% nutrients (mean ± s.e.m.). (A) The proportion of oocytes that cleaved and the proportions of cleaved embryos that formed blastocysts or initiated hatching on Day 7. (B) The numbers of cells in the trophectoderm (TE) and inner cell mass (ICM), as well as the total number of cells in the blastocyst (TE + ICM). Asterisks indicate treatments that were significantly different (*P* < 0.05) from the control (100%). Statistical trends (*P* < 0.10) are noted with #.

Treatment effects were evaluated with 1173 oocytes (292–295 oocytes, 168–177 cleaved embryos per treatment). The concentrations of nutrients present in the medium did not affect (*P* > 0.05) the proportion of oocytes that cleaved (57.5–60.0%) or the proportions of cleaved embryos that formed a blastocyst (35.6–45.2%) or initiated hatching (16.3–24.9%; Fig. 1). Reducing nutrient concentrations from 100 to 75% or 50% did not affect (*P* > 0.05) the numbers of cells within the TE or ICM of blastocysts (25–37 blastocysts per treatment), but the total number of cells in the blastocysts (126.8 ± 11.0) and the number of TE cells (104.7 ± 9.6 cells) were reduced (P<0.05) when embryos were cultured with 25% nutrients compared to those cultured with 100% nutrients (187.3 ± 11.8 and 154.8 ± 10.8, respectively; Fig. 1).

### Experiment 2: 100, 25, 12.5, and 6.25% nutrients

**Figure 2:**
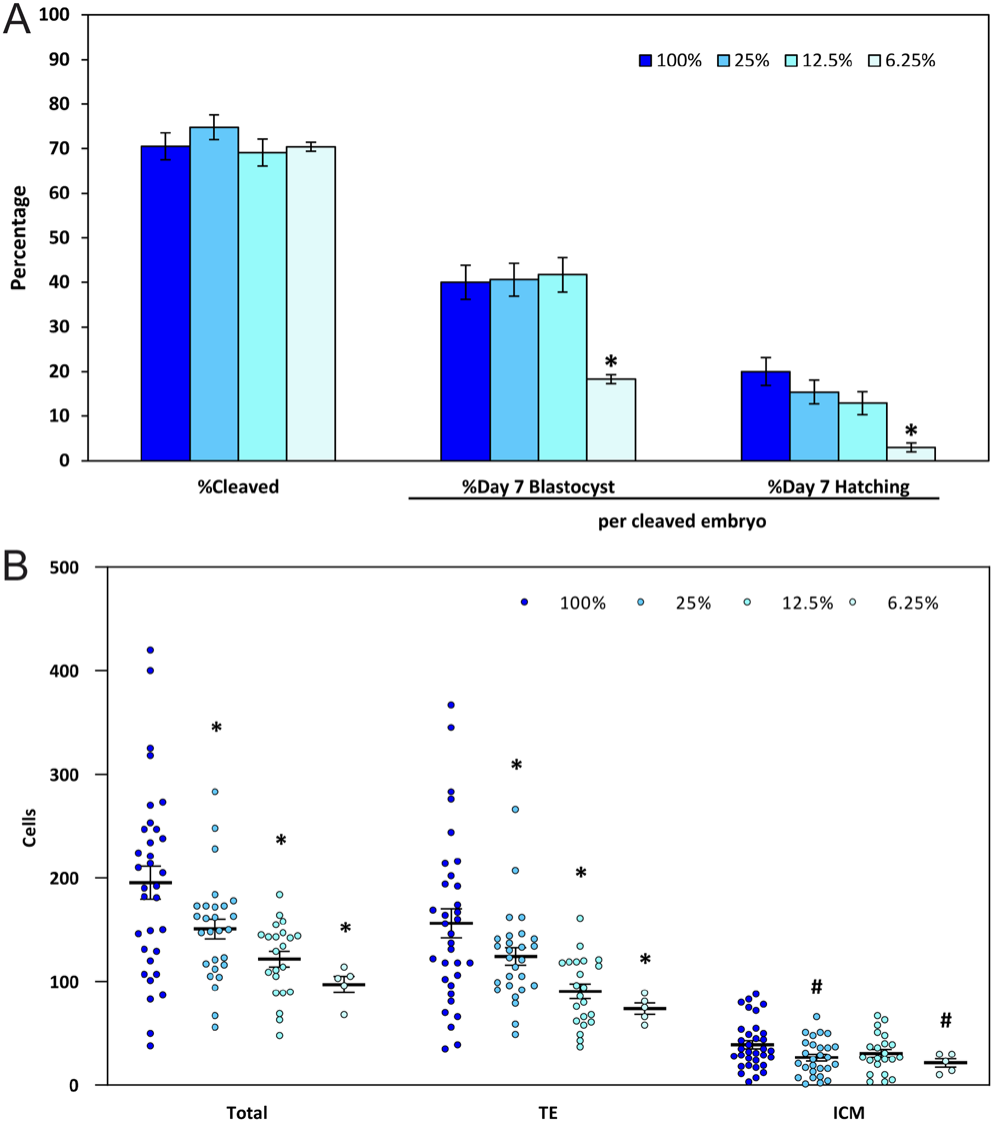
Development of embryos following culture in media containing 100, 25, 12.5, or 6.25% nutrients (mean ± s.e.m.). (A) The proportion of oocytes that cleaved and the proportions of cleaved embryos that formed blastocysts or initiated hatching on Day 7. (B) The numbers of cells in the trophectoderm (TE) and inner cell mass (ICM), as well as the total number of cells in the blastocyst (TE + ICM). Asterisks indicate treatments that were significantly different (*P* < 0.05) from the control (100%). Statistical trends (*P* < 0.10) are noted with #.

Treatment effects were evaluated with 937 oocytes (233–236 oocytes, 163–175 cleaved embryos per treatment). Cleavage (69.1–74.8%) was not affected (*P* > 0.05) by reducing the concentrations of the nutrients in the medium (Fig. 2). Culturing embryos with 25 or 12.5% nutrients did not affect (*P* > 0.05) blastocyst formation or hatching. However, when nutrients were further reduced to 6.25% both the proportion of embryos forming a blastocyst (18.3 ± 3.0) and hatching (3.0 ± 1.3%) were reduced (*P* < 0.05) compared to embryos cultured with 100% nutrients (40.0 ± 3.8% and 20.0 ± 3.1%, respectively; Fig. 2). All treatments with reduced nutrient concentrations produced blastocysts (22–33 blastocysts per treatment, except for 6.25%, *n* = 5) with fewer (*P* < 0.05) total cells and fewer (*P* < 0.05) cells in the TE (Fig. 2).

### Experiment 3: Cryosurvival

**Figure 3:**
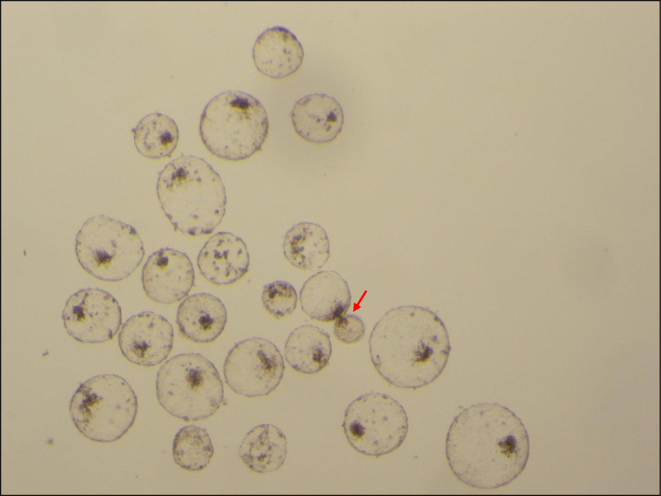
Hatched and hatching (arrow) blastocysts 48 h after warming and culture. Embryos were cultured in the presence of 25% nutrients and vitrified as expanded and early hatching blastocysts.

Expanded and early hatching blastocysts cultured with 100% (*n* = 28, 14 expanded and 14 hatching) or 25% (*n* = 23, 11 expanded and 12 hatching) nutrients were vitrified, warmed, and cultured. All embryos from both treatments survived and re-expanded after warming. All of the embryos cultured with 100% nutrients and 22 of 23 (95.7%) of the embryos cultured with 25% nutrients had completely hatched by 48 h post-warming (Fig. 3).

### Experiment 4: Role of fatty acid oxidation

**Figure 4:**
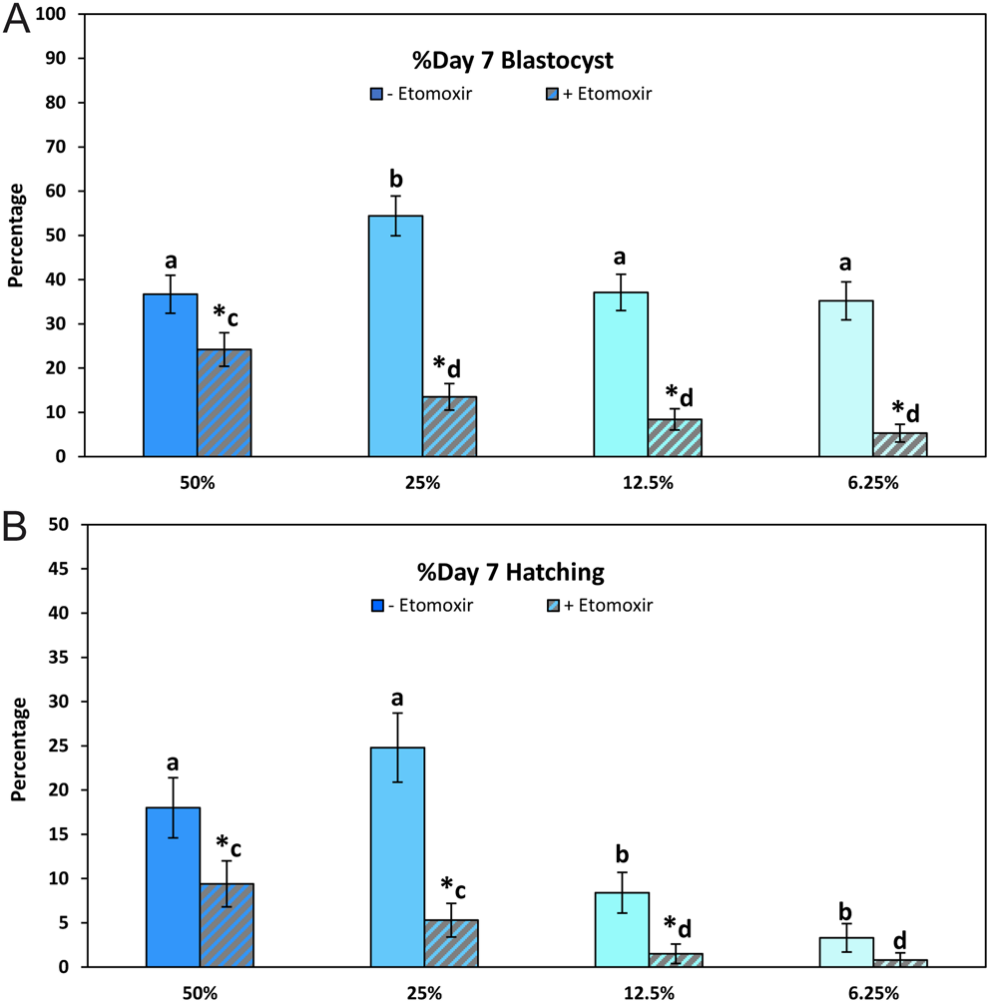
Blastocyst formation (A) and hatching (B) of embryos (mean ± s.e.m.) cultured with 50, 25, 12.5, or 6.25% nutrients in the absence (solid bars) or presence (striped bars) of etomoxir (50 µM), an inhibitor of fatty acid oxidation. Asterisks indicate a significant (*P* < 0.05) effect of etomoxir within the same concentration of nutrients. Different letters indicate a significant (*P* < 0.05) difference between nutrient concentrations for embryos cultured in the absence (a,b) or presence (c,d) of etomoxir.

To determine the role of endogenous lipid metabolism in the response of embryos to reduced nutrient conditions, embryos (1476 oocytes; 183–187 oocytes, 122–143 cleaved embryos per treatment) were cultured in media containing 50, 25, 12.5, and 6.25% nutrients in the presence or absence of 50 µM etomoxir for the entire culture period. The concentration of nutrients in the medium or the presence of etomoxir did not affect (*P* > 0.05) the proportion of oocytes that cleaved (66.8–76.9%). Etomoxir reduced (*P* < 0.05) blastocyst formation at all nutrient concentrations tested (Fig. 4). In addition, fewer (*P* < 0.05) blastocysts were produced when etomoxir was present in media with 25, 12.5, or 6.25% nutrients (≤13.9%) compared to the medium with 50% nutrients with etomoxir (24.3 ± 5.5%) (Fig. 4). Etomoxir reduced (*P* < 0.05) the proportion of hatching blastocysts when the medium contained 50, 25, or 12.5% nutrients, but not (*P* > 0.05) in media with 6.25% nutrients where hatching was low (<5%) regardless of whether etomoxir was present or not (Fig. 4).

### Experiment 5: Effects of nutrient dilution on blastocyst gene expression

**Figure 5:**
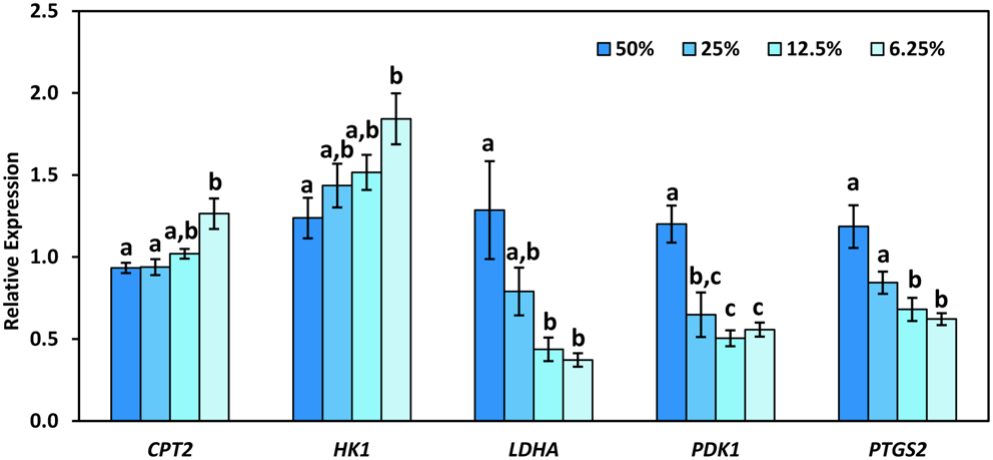
Expression of carnitine palmitoyltransferase 2 (*CPT2*), hexokinase 1 (*HK1*), lactate dehydrogenase A (*LDHA*), and pyruvate dehydrogenase kinase 1 (*PDK1*), and prostaglandin-endoperoxide synthase 2 (*PTGS2*) normalized against 18S ribosomal RNA (*RN18S1*) in blastocysts cultured with 50, 25, 12.5, or 6.25% nutrients. Different letters (a,b,c) indicate a significant (*P* < 0.05) difference in expression between nutrient concentrations.

To determine whether blastocysts differently modulated metabolic pathways in the reduced nutrient media (≤50%), we analyzed the expression of several metabolic genes. Reducing the nutrient composition of the medium to 6.25% increased (*P* < 0.05) expression of *HK1* and *CPT2* (Fig. 5). Expression of *LDHA* and *PTGS2* were decreased (*P* < 0.05) when nutrient concentrations were reduced to 12.5 or 6.25% (Fig. 5). Expression of *PDK1* was decreased (*P* < 0.05) at all nutrient concentrations below 50% (Fig. 5).

### Experiment 6: Effects of nutrient dilution on protein activation

**Figure 6:**
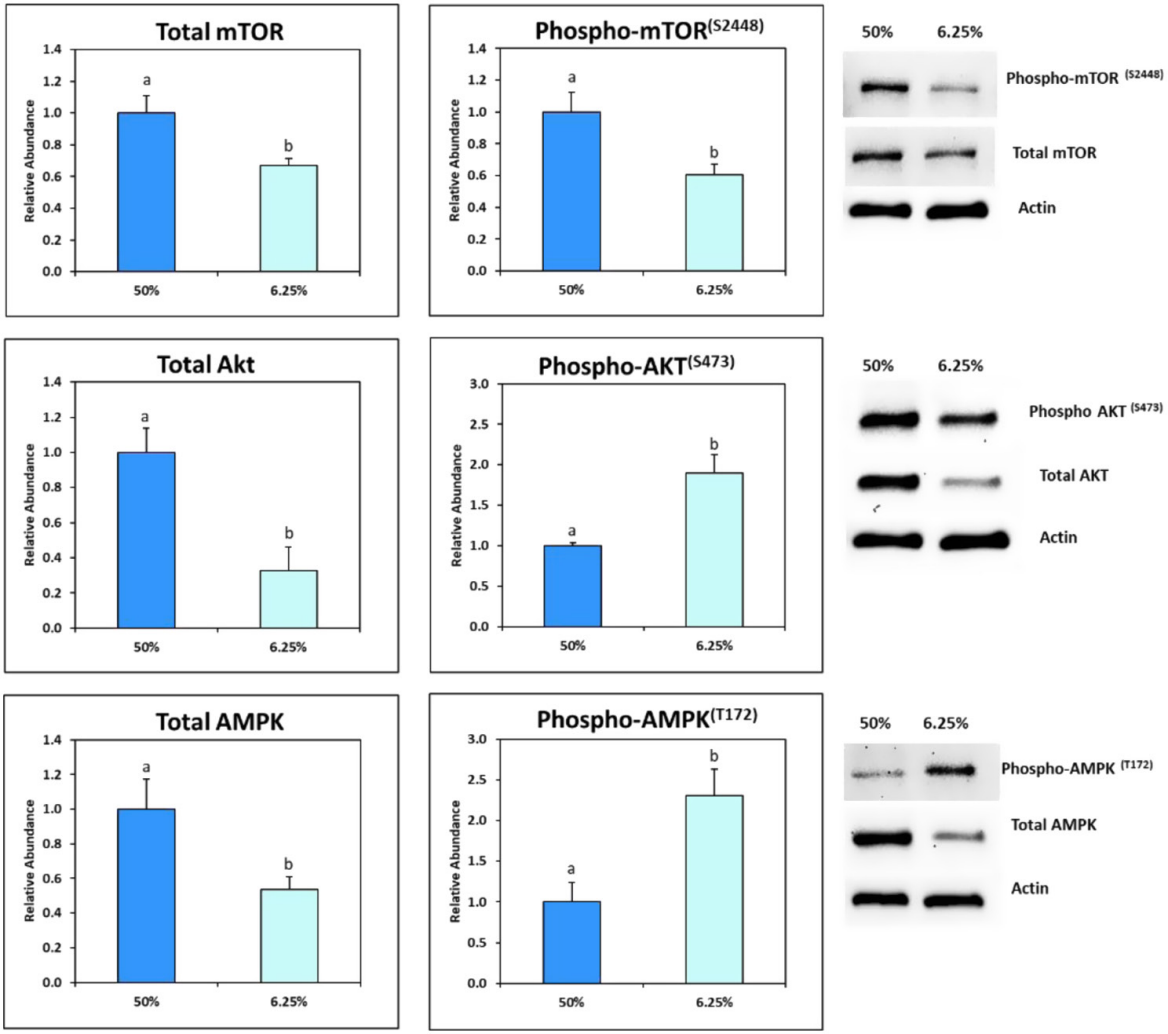
Western blot analysis of total and phosphorylated (phospho-) forms of mammalian target of rapamycin (mTOR and mTOR^(Ser2448)^), adenosine monophosphate-activated protein kinase (AMPK and AMPK^(T172)^), and protein kinase B (AKT and AKT^(S473)^). Total protein abundance was expressed relative to actin and phosphorylation level was determined based on the ratio of phosphorylated and total protein abundance in each sample. Different letters (a,b) indicate significant (*P* < 0.05) differences between blastocysts cultured with 6.25% nutrients compared to blastocysts cultured with 50% nutrients.

Western blot analysis was used to determine the activity of AMPK, AKT and mTOR pathways in bovine blastocysts produced in reduced nutrient conditions based on the levels of stimulatory phosphorylation. Blastocysts cultured with 6.25% nutrients had increased (*P* < 0.05) amounts of phosphorylated/active Akt and AMPK, but less (*P* < 0.05) phosphorylated/active mTOR, compared to embryos cultured with 50% nutrients (Fig. 6).

## Discussion

Bovine embryos were found to be remarkably resilient to reductions in the concentrations of exogenous nutrients present in the culture medium. A medium containing 50% of the nutrients present in our standard medium supported the development of embryos that were not different from controls (100% nutrients) with respect to blastocyst formation, hatching, and the number of cells present in the TE and ICM. A further reduction in nutrients to 25% decreased the number of TE cells in resulting blastocysts, but the proportions of embryos forming blastocysts and initiating hatching were unaffected. Although embryo transfer experiments were not conducted to evaluate embryonic viability, blastocysts produced with 25% nutrients were able to survive (re-expansion and hatching) vitrification and warming in similar proportions as embryos produced with 100% nutrients. Even when nutrients were reduced to 6.25%, ~20% of embryos were still capable of developing to the blastocyst stage, but differences in gene expression and protein activity were observed suggesting the viability of these blastocysts may have been compromised. Importantly, the nutrient composition of our control medium (100%) was similar to media used by other laboratories, as well as the reported composition of nutrients in bovine oviductal and uterine fluid (Tables 1 and 2) (Hugentobler *et al.* 2007, 2008). The density of embryos in the current study (5–10 embryos per 20 µL) was also similar to those used by other laboratories (Liu & Foote 1996, Gandhi *et al.* 2000, Sutton-McDowall *et al.* 2012, Tribulo *et al.* 2019). Although the exact proportion of nutrient dilution that can be tolerated by bovine embryos will depend on the composition of the original medium, the number of embryos cultured per volume of medium, and the duration of culture, the results of our study indicate that the amount of nutrients provided to bovine embryos in many culture protocols far exceed the amounts needed for development. Development of bovine embryos to the four-cell stage has been demonstrated in the absence of exogenous nutrients (Sutton-McDowall *et al.* 2012), but, to the best of our knowledge, this is the first report of bovine blastocyst production in media containing total nutrient concentrations as low as those found in our 12.5 and/or 6.25% dilutions (Tables 1 and 2) (Tervit *et al.* 1972, Takahashi & First 1992).

The ability of bovine embryos to develop in the presence of reduced concentrations of exogenous nutrients suggests that either the embryos need very little energy to develop to the blastocyst stage or they are capable of utilizing endogenous energy stores to support development. When FAO was inhibited by culturing embryos with an inhibitor of carnitine palmitoyl transferase 1 (etomoxir), blastocyst formation and hatching decreased regardless of the concentrations of nutrients in the medium, indicating FAO is active in bovine embryos. Inhibition of FAO in embryos cultured with 50% nutrients reduced blastocyst formation by ~13%, similar to the results of Ferguson and Leese (2006) using an alternative FAO inhibitor in a culture medium with conventional nutrient concentrations. In contrast, inhibition of FAO in embryos cultured with 6.25% nutrients reduced blastocyst formation by ~30%. In addition, fewer embryos cultured with ≤25% nutrients and etomoxir developed to the blastocyst stage compared to those cultured with 50% nutrients and etomoxir. The difference in blastocyst development as nutrient concentrations decrease in the presence of etomoxir, as well as the increased expression of *Cpt2* in embryos cultured with 6.25% nutrients, suggests an increased reliance on FAO as nutrient concentrations are decreased. Similarly, bovine embryos can develop into morulae (~20%) if carnitine (essential co-factor for FAO, 5 mM) is present in a medium lacking exogenous nutrients (Sutton-McDowall *et al.* 2012). These findings support the ability of the bovine embryo to utilize endogenous fatty acids when exogenous nutrients are limited, similar to what has been reported for several cancer cell lines (Buzzai *et al.* 2005, Zaugg *et al.* 2011). Also, both our study and the study of Sutton-McDowall *et al.* ( 2012) utilized BSA (2.5–4.0 mg/mL) in the culture media. Despite reports that embryos can take up albumin from the medium (Thompson *et al.* 1998, Sun *et al.* 2014), the nutrients provided by the albumin are apparently not sufficient to make up for severe deficiencies in the availability of extracellular carbohydrates and amino acids.

The ability of bovine embryos to metabolize endogenous fatty acids may explain the different responses of murine and bovine embryos to similarly reduced concentrations of nutrients (Ermisch *et al.* 2020). Bovine oocytes are known to contain higher concentrations of lipids than murine oocytes (Genicot *et al.* 2005). Inhibition of FAO in bovine oocytes and embryos during *in vitro* maturation or embryo culture, respectively, has more dramatic negative effects than similar concentrations of the same inhibitors on murine oocytes and embryos, suggesting that lipids are being used in larger quantities in bovine embryos (Hewitson *et al.* 1996, Ferguson & Leese 2006, Paczkowski *et al.* 2013). However, in both species FAO is dependent on the presence of carnitine (Dunning *et al.* 2010, Sutton-McDowall *et al.* 2012). It is unclear if bovine embryos have larger stores of carnitine or an enhanced ability to synthesize carnitine that would facilitate their ability to switch to FAO when other nutrients are in limited supply.

Analysis of gene expression suggested that reduced nutrient concentrations led to alterations in carbohydrate metabolism in bovine blastocysts. In standard culture conditions, the metabolic profile of blastocysts is characterized by active glycolysis, even in the presence of oxygen, with the resulting pyruvate converted to lactate rather than entering the tri-carboxylic acid (TCA) cycle (Krisher & Prather 2012). Glucose is also metabolized through the pentose phosphate pathway to produce ribose 5-phosphate and NADPH for cell proliferation. In this scenario, amino acids and fatty acids become the primary sources for ATP production via the TCA cycle (Krisher & Prather 2012). Many cancer cells also exhibit this pattern of metabolism, known as the Warburg Effect (WE) (Courtnay *et al.* 2015). However, when nutrients are limited, the metabolic response is specific to the type of cancer. For some cancer cell types, the WE continues to persist when nutrients are limited, while other cancers exhibit an ‘anti-WE’ when exposed to reduced nutrients, characterized by reduced glucose uptake, reduced glycolysis, and increased oxygen consumption (Wu *et al.* 2013, Bianchi *et al.* 2015). Although gene expression does not necessarily indicate enzymatic activity, bovine embryos exhibited an upregulation of both glycolysis and pyruvate oxidation that resembles both the WE and the anti-WE, respectively. Increased expression of *HK1* would allow an increased flux of glucose through glycolysis, with decreased *LDHA* (less pyruvate converted to lactate) and *PDK1* (less inhibition of pyruvate dehydrogenase) allowing more of the pyruvate produced by glycolysis to enter the TCA cycle. Combined with the increase in FAO discussed above, bovine embryos appear to be upregulating most, if not all, major pathways for ATP production to cope with the extreme restriction in nutrient availability (6.25%).

Metabolic changes in response to the availability of extracellular nutrients are often mediated by interactions between AMPK and mTOR. When nutrients are limited, intracellular concentrations of ATP decrease and concentrations of AMP increase, leading to the phosphorylation (threonine 172) and activation of AMPK (Hardie *et al.* 2012). Active AMPK increases ATP-generating metabolism, including glucose uptake, glycolysis, and FAO (Zaugg *et al.* 2011, Zhao *et al.* 2017). AMPK also reduces further ATP consumption by inhibiting the action of mTOR (Hardie *et al.* 2012, Zhao *et al.* 2017). Consistent with these patterns, the activity of AMPK was increased and the activity of mTOR was decreased in blastocysts cultured with reduced nutrients. AKT also participates in the cell’s response to nutrient availability. When nutrients are available, AKT is active and promotes glucose uptake and glycolysis (Elstrom *et al.* 2004, Zhao *et al.* 2017). However, when glucose is limited, the activity of AKT is cell-type specific (Bianchi *et al.* 2015, He *et al.* 2016). Our results demonstrate that the activity of AKT was increased when bovine blastocysts were cultured with reduced nutrients. Limited glucose availability and pharmacological activation of AMPK also led to increased AKT activity in a murine trophoblast stem cell line (Louden *et al.* 2008). Collectively, these results suggest an important role of AMPK, mTOR, and AKT pathways in shifting the metabolic regulation of embryos to cope with metabolic stress caused by reduced nutrient availability.

In addition to changes in cellular metabolism, reduced concentrations of nutrients (6.25%) were also associated with a decreased incidence of hatching. It is possible that the reduced cell numbers of the blastocysts, combined with reduced nutrients for ATP production, may have been insufficient to generate the force needed for blastocyst expansion and hatching from the zona pellucida (Leonavicius *et al.* 2018). However, another possible mechanism for hatching may be embryo-derived prostaglandins, as has been reported in murine blastocysts (Huang *et al.* 2004). Expression of cyclooxygenase 2 (COX2) and prostaglandin E synthase (PGES) is increased during bovine blastocyst formation and dependent on blastocyst quality (Saint-Dizier *et al.* 2011). Similarly, good quality bovine embryos with a higher incidence of hatching secrete more prostaglandin E2 into the culture medium (Boruszewska *et al.* 2019). In our study, blastocysts cultured under reduced nutrient conditions exhibited reduced expression of *PTGS2* (also known as *COX2*) and had a decreased incidence of hatching, supporting a possible role for prostaglandins in the hatching of bovine blastocysts.

The results of this study and our recent report in murine embryos (Ermisch *et al.* 2020) highlight the extraordinary adaptability of preimplantation embryos to extracellular nutrient availability. The degree of nutrient reduction that can be tolerated by the embryo will be dependent on the specific culture medium used, the culture volume, the number of embryos present, and whether or not the embryos are transferred to fresh medium at any point of the culture period. In our laboratory, murine and bovine embryos are cultured in a sequential medium system (medium renewal on day 2 or 3 of culture) in groups of 5–10 embryos per 20 µL. Under these conditions, nutrient concentrations could be reduced by 50% (below reported *in vivo* concentrations (Harris *et al.* 2005, Hugentobler *et al.* 2007, 2008)) with no effect on blastocyst formation, hatching, or cell allocation. If the nutrients in the culture environment are present at ≥2× the minimum required concentrations, it is perhaps not surprising that it is has been historically difficult to identify preferred substrates, critical metabolic pathways, or a single, optimal combination of nutrients for use in embryo culture media. For bovine embryos, nutrient concentrations in the medium could be reduced to as little as 6.25% (<1 mM total nutrients), and still support limited blastocyst formation and hatching. However, these dramatic changes in nutrient availability were also associated with altered gene expression and protein phosphorylation/activation, which may have long-term effects on the offspring. Before reduced nutrient media are used to produce embryos for agricultural or biomedical applications, additional testing, including embryo transfers, would be necessary. However, these novel culture conditions may provide a sensitive experimental system to identify specific substrates and metabolic pathways that are critical for pre- and post-implantation development. For example, Sutton-McDowall *et al.* (2012) demonstrated a significant effect of carnitine on bovine embryo development in the absence of carbohydrates or amino acids, while the effects of carnitine on the development of bovine embryos in a more typical, nutrient-rich medium have been inconsistent (Takahashi *et al.* 2013, Zolini *et al.* 2019). Similarly, it may be possible to produce blastocysts with a specific metabolic phenotype (e.g., increased AMPK activity and enhanced FAO) by altering nutrient concentrations (e.g., ≤25% nutrients) rather than using pharmacological stimulators or inhibitors (De La Torre-Sanchez *et al.* 2006, Barcelo-Fimbres & Seidel 2007, Louden *et al.* 2008, Calder *et al.* 2017). Additional work with embryos cultured in reduced nutrient conditions will be useful in expanding our understanding of embryonic metabolism and could make significant contributions to the continued evolution of culture media.

## Declaration of interest

The authors declare that there is no conflict of interest that could be perceived as prejudicing the impartiality of the research reported.

## Author contribution statement

J R H was responsible for experimental design, embryo culture experiments, and manuscript preparation. S R completed the western blot experiments and assisted with manuscript preparation. R P and N S completed the gene expression experiments and assisted with manuscript preparation. A E assisted with all aspects of the embryo culture experiments and manuscript preparation. W B S assisted with experimental design and provided financial support. R L K provided guidance on experimental design, data interpretation, and manuscript preparation.
